# Evaluation of Fibroblast Viability Seeded on Acellular Human Amniotic Membrane

**DOI:** 10.1155/2021/5597758

**Published:** 2021-05-24

**Authors:** Hamideh Moravvej, Hamed Memariani, Mojtaba Memariani, Maryam Kabir-Salmani, Alireza Shoae-Hassani, Fahimeh Abdollahimajd

**Affiliations:** ^1^Skin Research Center, Shahid Beheshti University of Medical Sciences, Tehran, Iran; ^2^Biomaterials and Tissue Engineering Department, Stem Cell Division, National Institute of Genetic Engineering and Biotechnology, Tehran, Iran; ^3^Stem Cell and Regenerative Medicine Research Center, Iran University of Medical Sciences, Tehran, Iran; ^4^Clinical Research Development Unit, Shohada-e Tajrish Hospital, Shahid Beheshti University of Medical Sciences, Tehran, Iran

## Abstract

**Background:**

Investigating the viability and proliferative rates of fibroblast cells on human amniotic membrane (HAM) as a scaffold will be an important subject for further research. The aim of this study was to assess the fibroblast viability seeded on acellular HAM, since foreskin neonatal allogenic fibroblasts seeded on HAM accelerate the wound healing process.

**Methods:**

Fibroblasts were retrieved from the foreskin of a genetically healthy male infant, and we exploited AM of healthy term neonates to prepare the amniotic scaffold for fibroblast transfer. After cell culture, preparation of acellular HAM, and seeding of cells on HAM based on the protocol, different methods including 3-(4,5-dimethylthiazol-2-yl)-2,5-diphenyltetrazolium bromide (MTT), 4′,6-Diamidino-2-phenylindole dihydrochloride (DAPI), and propidium iodide (PI) staining were employed for assessment of fibroblast viability on HAM.

**Results:**

Based on the results obtained from the DAPI and PI staining, the percentage of viable cells in the former staining was clearly higher than that of the dead cells in the latter one. The results of DAPI and PI staining were in accordance with the findings of MTT assay, confirming that fibroblasts were viable and even proliferate on HAM.

**Conclusion:**

Our findings showed the viability of fibroblasts seeded on the acellular HAM using MTT assay, DAPI, and PI staining; however, this study had some limitations. It would be an interesting subject for future research to compare the viability and proliferation rate of fibroblasts seeded on both cellular and acellular HAM.

## 1. Introduction

As one of the major public health problems, chronic wounds lead to increased morbidity, disability, and risk of mortality, imposing a considerable financial burden on both patients and healthcare system [1, 2]. As a result, a great deal of attention has been devoted to wound care management, in particular different biomaterial scaffolds and cell sources. Although there are no standardized treatment guidelines in this area, the efficacy of commercialized products such as bioengineered skin substitutes, topical growth factors, and stromal matrices needs to be determined [2].

Over the past years, a growing number of studies have provided us major improvements for treatment of different wounds. Recent innovative skin grafts using natural or synthetic scaffolds employing stem cells emerge as a novel therapeutic solution. One of the most widely used biomaterials for the abovementioned purpose is human amniotic membrane (HAM) [3].

The amniotic membrane is a thin, semitransparent, and multilayered membrane with a complex structure. It is composed of a monolayer of metabolically active epithelium, a basement membrane, a compact layer, a fibroblast layer, and a spongy layer [4]. A diverse array of growth factors such as transforming growth factor-*β* and epidermal growth factor as well as cytokines including interleukin- (IL-) 1 receptor antagonist, IL-6, 8, and 10 have been shown to produce by the epithelium and stromal matrix [5]. Low immunogenicity, good biocompatibility, and high affinity to human stem cells are among the reasons that make HAM as an attractive candidate for wound dressing [6, 7]. Although HAM has a long history in treatment of wounds as a biological dressing, recent understanding of its appropriate adhesion to stem cells has made this biomaterial a suitable skin substitute [8]. Foreskin neonatal allogenic fibroblasts seeded on such scaffolds accelerate the wound healing and enhance its proregenerative capacity [6, 9].

Currently, the application of these cells is the subject of many contemporary studies [6, 10]. It has been shown that human fibroblasts cultured on acellular HAM exhibit a well-defined spindle-shaped morphology together with fast proliferation [6]. On the other hand, collagen expressed by fibroblasts and angiogenesis induced by mesenchymal stem cells are essential for wound healing process especially in diabetic wounds [11].

In spite of a variety of protocols for processing of cell seeding on HAM, they still share common procedures including isolation, culturing, and seeding of the cells on the associated material scaffold. It is worthwhile to note that the time-consuming process of cell culture along with repeated trypsinization needed for obtaining fibroblasts to seed on HAM may lead to decreased proliferative ability and cell viability [6]. As a consequence, it is necessary to consider such factors when designing the protocols of cell processing for seeding on HAM.

3-(4,5-Dimethylthiazol-2-yl)-2,5-diphenyltetrazolium bromide (MTT), 4′,6-diamidino-2-phenylindole dihydrochloride (DAPI), and propidium iodide (PI) staining can be used to evaluate cell viability. The MTT assay is a colorimetric test for measurement of the metabolic activity of cells. In this context, NAD (P)H-dependent cellular oxidoreductase enzymes indicate the number of viable cells [12]. DAPI is a blue fluorescent nucleic acid stain for identification of cell cycle and specifically stains viable nuclei [13]. PI is a red fluorescent intercalating substance for staining dead cells among other cells [14].

We were able to find only one study that assessed the effect of adding human amniotic membrane-derived mesenchymal stem cells (HAM-MSCs) on the viability and proliferative ability of the fibroblasts and keratinocytes [15]; so in this study, we aimed to assess the fibroblast viability seeded on human amniotic membrane using MTT assay, DAPI, and PI staining.

## 2. Materials and Methods

### 2.1. Cell Culture, Preparation of HAM, and Seeding of Cells on HAM

We prepared decellularized amniotic membranes according to our previous research. In brief, in order to prepare the amniotic scaffold for fibroblast transfer, we exploited amniotic membranes of healthy term neonates who were born through caesarean section. The AM was stored at -80°C until further use. Frozen AMs were thawed at room temperature (RT), washed three times with phosphate buffer saline (PBS), and then cut into small pieces. The AMs were exposed to three freeze–thaw cycles which were performed by freezing at 80°C and thawing in distilled water at 37°C. Afterward, tissues were immersed in trypsin–EDTA solution at 4°C overnight. At the final stage, trypsin was neutralized with DMEM and the cells were separated from membranes effectively. This process was carried out at 4°C to reduce degradation of extracellular matrix (ECM) protein. After three washes with PBS, the scaffold was stored at -80°C for up to 3 months [9].

**Figure 1:**
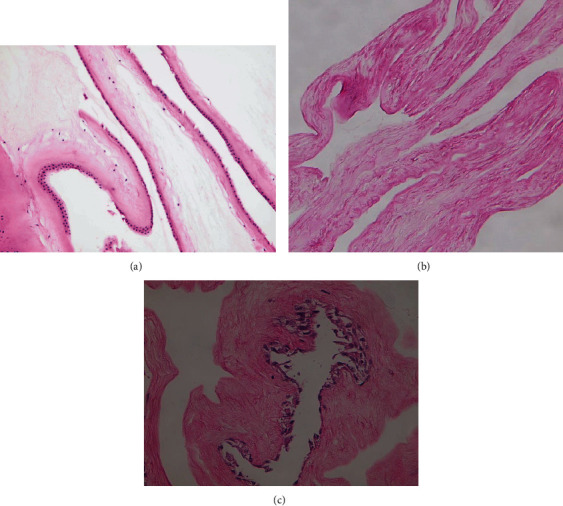
(a) The histology of an intact human amniotic membrane; (b) the cells of the amniotic membrane have been scraped off; (c) the amniotic membrane with the seeded fibroblasts.

Fibroblasts were retrieved from the foreskin of a genetically healthy male infant [9]. The study protocol was prepared in accordance with the Declaration of Helsinki, with the approval of the Ethics Board Committee on Medical Research of our institution. The protocol of the cell culture, preparation of HAM, and seeding of cells on HAM (Figure 1) has been explained in full details in the previous published paper [16].

### 2.2. Evaluation of Cell Viability on HAM

In order to assess viability of fibroblasts on HAM, different methods including MTT, DAPI, and propidium iodide (PI) staining were employed.

#### 2.2.1. MTT Assay

The MTT (3-(4,5-dimethylthiazol-2-yl)-2,5-diphenyltetrazolium bromide, Sigma-Aldrich; Merck KGaA, Darmstadt, Germany) colorimetric staining assay was performed according to the manufacturer's guidelines. All wells were incubated with 1 ml of MTT (1 mg/ml) for 5 h at 37°C with 5% CO_2_. The MTT was then removed, and 1 ml of isopropanol was added, followed by another incubation period of 1 h at 37°C with 5% CO_2_. Color changes due to the conversion of MTT to blue formazan dye were measured using a multiplate reader (model 680 Bio-Rad, USA) at a wavelength of 570 nm. The MTT was added to the control and experimental groups, including Dulbecco's modified Eagle's medium (DMEM) without cells (as a negative control group), amniotic membrane without cell, fibroblast cells, and amniotic membrane with seeded fibroblast cells (FAM).

#### 2.2.2. DAPI Staining

Nuclei of cells cultured on the AM were stained with DAPI (Sigma-Aldrich; US) and viewed using the immune-fluorescent microscopy. Briefly, cell-seeded AMs were fixed in 4% paraformaldehyde (Merck; US) for 30 min at 4°C and then embedded in optimal cutting temperature compound (OCT, Sigma; US) and stored at −80°C. The AMs were cryosectioned in 6 *μ*m thickness and fixed in acetone (Merck; Germany) at −20°C for 15 min. Afterwards, they were incubated in 1% Triton X-100 (Sigma; US) for 10 min. Following three rinses with phosphate buffer saline (PBS, Gibco; UK), each for 5 min, they were preincubated with 2% bovine serum albumin (BSA, Sigma; US). Finally, after rinsing with PBS, the DAPI-stained nuclei were revealed as light blue granular organelles.

#### 2.2.3. PI Staining

Regarding PI staining, several steps including fixation, washing, and permeabilizing were performed. Since PI binds to RNA, it is essential to treat culture with nucleases to distinguish between RNA and DNA staining. Therefore, RNase A (0.2-0.5 mg/ml) (GeneAll®) was added to the preparations and incubated for 1 h at 37°C. Following washing with PBS, PI solution (10 *μ*g/ml) (Roti®-Mount FluorCare; Carl Roth GmbH & Co. KG, Germany) was added to cover the amniotic membrane scaffold in the darkness at 4°C until analysis through fluorescence microscopy (Olympus BX60, IX70 Olympus Optical Co., Tokyo, Japan), with an excitation and emission wavelength of 540 and 590 nm, respectively.

### 2.3. Statistical Methods

The statistical software SPSS 16.0.0 (SPSS Inc. Chicago, IL, USA) was used for all data analyses. *P* values less than 0.05 were considered statistically significant.

## 3. Results

**Figure 2:**
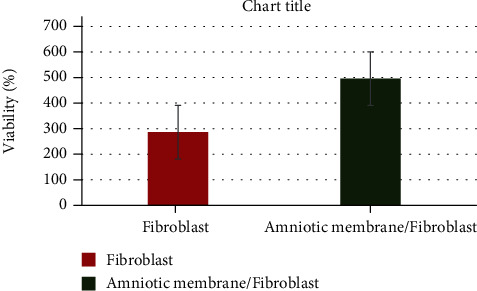
Evaluation of the cytotoxic effect of decellularized amniotic membrane on fibroblast cells after seeding. The cytotoxic effect was determined based on cell viability after seeding on the amniotic membrane for 48 h in the MTT test. The results are presented as mean ± SEM (*n* = 6). ^∗∗∗∗^*P* < 0.00001, *vs*. control.

Figure 1 shows the histology of an intact HAM, decellularized HAM, and the AM with the seeded fibroblasts. MTT assay indicated a significant increase in viability when fibroblast cells were cultured on the amniotic membrane (*P* < 0.05) compared to control (Figure 2).

**Figure 3:**
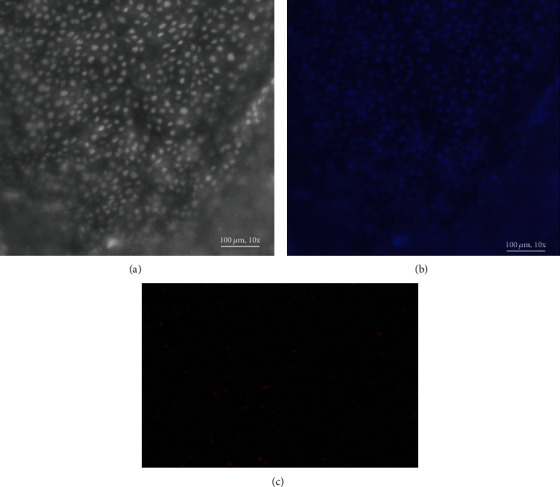
(a, b) Viable nuclei were visualized with DAPI staining; (c) nonviable cells were shown using PI staining (red fluorescence).

There was a statistically significant increase in cell viability in the sample group (amniotic membrane seeded with fibroblast cells) compared to the control groups (fibroblast cells) (*P* < 0.05; Figure 2). Based on the results obtained from the DAPI and PI staining, the percentage of viable cells in the former staining was clearly higher than the dead cells in the latter one (Figure 3). Taken together, the results of DAPI and PI staining were in accordance with the findings of MTT assay, confirming that fibroblasts were still viable and even proliferate on HAM.

## 4. Discussion

Our findings based on the MTT assay, DAPI, and PI staining revealed the viability and proliferative ability of the fibroblasts seeded on HAM. Indeed, AM is an ideal choice for therapeutic applications owing to limitless availability, convenience in procurement, relative cost-effectiveness, and low immunogenicity. Antifibrotic, anti-inflammatory, anticancer, antimicrobial, wound healing, and scaffold-like properties of AM have been well proven in the literature; this is due in part to producing different cytokines and growth factors by amniotic-derived epithelial cells and mesenchymal stromal cells of amniotic membranes [17, 18]. Doubtlessly, AM has various clinical applications in the several fields of medicine including dermatology, ophthalmology, surgery, orthopedics, and urology.

Biomaterials are generally referred to as scaffolds, constructs, or matrices that enable cells to adhere, proliferate, and differentiate, ultimately resulting in the formation of a new tissue. Several considerations must be kept in mind when using scaffolds in tissue engineering. These include biocompatibility, biodegradability, mechanical properties, scaffold architecture, and manufacturing technology [19]. Based on the proposed usage, scaffolds can be categorized as either natural or synthetic and degradable or nondegradable [20]. As a natural material, AM has attracted huge attention in the field of tissue engineering and regenerative medicine due to its good biocompatibility and favorable mechanical features such as elasticity, flexibility, permeability, plasticity, resorbability, and stability [21]. The amniotic membrane can serve as a scaffold for not only proliferation but also differentiation of cells owing to the presence of extracellular matrix constituents such as different types of collagen (i.e., I, II, III, IV, V, and VII), elastin, fibronectin, hyaluronic acid, laminin, and nidogen [22]. Evidence abounds in literature with regard to the use of HAM in treating various skin diseases. In a study conducted by Khazaei et al. [23], fresh HAM was effective in improving rabbit perianal surgery wounds. Consistent with these results, decellularized HAM hastened the process of the wound self-healing in full-thickness skin defects of rats [24]. When used to cover burn wounds, HAM led to rapid epithelialization, wound healing, and reduced pain [25]. Moreover, a proof-of-concept study demonstrated the usefulness of HAM in treating chronic wounds in patients with epidermolysis bullosa [26]. Some studies have shown that the amniotic membrane transplantation in the acute phase of toxic epidermal necrolysis with ocular involvement preserves visual acuity [27].

Previous studies indicated that different cell lines are capable of adhering to and/or proliferating on decellularized HAM [9]. These are exemplified by bone marrow-derived mesenchymal stem cells (BM-MSC), adipose tissue-derived stem cells, fibroblasts, and keratinocytes [28]. As mentioned previously, foreskin neonatal allogenic fibroblast seeded on HAM accelerates the wound healing and enhances its proregenerative capacity. In a study conducted by Moravvej et al., two methodologies of cell therapy were assessed in patients with recessive dystrophic epidermolysis bullosa (RDEB); not only did direct injection of fibroblasts into wounds result in complete wound closure, but also fibroblast seeded on HAM promoted healing of RDEB wounds [16].

Although various studies have addressed the therapeutic effects of using stem cells and fibroblasts on different scaffolds, investigating the viability and proliferative rates of these cells on their respective scaffolds will be an important and interesting subject for further research [29]. Few studies have been conducted in this regard. In a study performed by Costa et al., the proliferative and osteogenic differentiation of MSCs seeded on a melt-based chitosan scaffold was evaluated using cell viability assay MTS. Their results indicated that these cells were viable even for 3 weeks after culture [30]. In another study, Ghiasi et al. compared viability and proliferation rates of adipose-derived stem cells on five different scaffolds including alginate, fibrin glue, poly lactic coglycolic acid, inactive, and active platelet-rich plasma (PRP) using some assays including MTT, analogue of stemness gene expression, and DNA content assay. The authors demonstrated that active PRP and alginate can serve as the most and least suitable scaffolds in terms of enhancing cell proliferation and maintaining cell viability, respectively [31].

Except for one study that assessed the effect of HAM-MSCs on keratinocytes and fibroblasts, we were unable to find a study that addressed the viability of fibroblasts seeded on HAM. In the mentioned study, Kitala et al. evaluated the influence of amniotic stem cells on a number of dermal and epidermal cells in various cell cycle phases as well as their angiogenesis induction capability. They showed that adding amniotic cells to both keratinocytes and fibroblast cultures accelerates directional migration by ≥40% while impairing their angiogenesis capability [15].

## 5. Conclusions

In our study, we demonstrated the viability of fibroblasts seeded on the acellular human amniotic membrane using MTT assay, DAPI, and PI staining; however, this study had some limitations. It is mandatory to investigate the number of cells and their proliferation in various cell cycle phases and DNA content assay; on the other hand, it would be an interesting subject for future research to compare the viability and proliferation rate of fibroblasts seeded on both cellular and acellular HAM that would enrich this paper.

## Data Availability

The data used to support the findings of this study are included within the article.

## Abbreviations

AM: Amniotic membrane
DAPI: 4′,6-Diamidino-2-phenylindole dihydrochloride
DMEM: Dulbecco's modified Eagle's medium
ECM: Extracellular matrix
HAM: Human amniotic membrane
IL: Interleukin
MSC: Mesenchymal stem cells
MTT: 3-(4,5-Dimethylthiazol-2-yl)-2,5-diphenyltetrazolium bromide
PBS: Phosphate buffer saline
PRP: Platelet-rich plasma
PI: Propidium iodide
RDEB: Recessive dystrophic epidermolysis bullosa.

## Additional Points

*What Is Already Known about This Topic?* (i) Low immunogenicity, good biocompatibility, and high affinity of amniotic membrane to human stem cells make it as an attractive candidate for wound dressing. *What Does This Study Add?* (i) The findings of MTT assay confirmed that fibroblasts were viable and even proliferate on human amniotic membrane; (ii) the percentage of viable cells in DAPI staining was clearly higher than the dead cells in PI staining; and (iii) future studies are needed to compare the viability of fibroblasts seeded on both cellular and acellular HAM.
